# Time-series maps of aboveground biomass in dipterocarps forests of Malaysia from PALSAR and PALSAR-2 polarimetric data

**DOI:** 10.1186/s13021-018-0108-2

**Published:** 2018-10-19

**Authors:** Hamdan Omar, Muhamad Afizzul Misman

**Affiliations:** Geoinformation Programme, Division of Forestry and Environment, Forest Research Institute Malaysia, 52109 Kepong, Selangor Malaysia

**Keywords:** Tropical forests, Biomass carbon, L-band SAR

## Abstract

**Background:**

Malaysia typically suffers from frequent cloud cover, hindering spatially consistent reporting of deforestation and forest degradation, which limits the accurate reporting of carbon loss and CO_2_ emissions for reducing emission from deforestation and forest degradation (REDD+) intervention. This study proposed an approach for accurate and consistent measurements of biomass carbon and CO_2_ emissions using a single L-band synthetic aperture radar (SAR) sensor system. A time-series analysis of aboveground biomass (AGB) using the PALSAR and PALSAR-2 systems addressed a number of critical questions that have not been previously answered. A series of PALSAR and PALSAR-2 mosaics over the years 2007, 2008, 2009, 2010, 2015 and 2016 were used to (i) map the forest cover, (ii) quantify the rate of forest loss, (iii) establish prediction equations for AGB, (iv) quantify the changes of carbon stocks and (v) estimate CO_2_ emissions (and removal) in the dipterocarps forests of Peninsular Malaysia.

**Results:**

This study found that the annual rate of deforestation within inland forests in Peninsular Malaysia was 0.38% year^−1^ and subsequently caused a carbon loss of approximately 9 million Mg C year^−1^, which is equal to emissions of 33 million Mg CO_2_ year^−1^, within the ten-year observation period. Spatially explicit maps of AGB over the dipterocarps forests in the entire Peninsular Malaysia were produced. The RMSE associated with the AGB estimation was approximately 117 Mg ha^−1^, which is equal to an error of 29.3% and thus an accuracy of approximately 70.7%.

**Conclusion:**

The PALSAR and PALSAR-2 systems offer a great opportunity for providing consistent data acquisition, cloud-free images and wall-to-wall coverage for monitoring since at least the past decade. We recommend the proposed method and findings of this study be considered for MRV in REDD+ implementation in Malaysia.

## Background

Global forests cover only approximately 26% of the Earth’s land mass, which is equal to approximately 38.5 million square kilometers. Despite the small composition, this area is shrinking and has decreased at approximately 13 million ha per year over the last few decades [1]. Forests, especially in the tropics, are suffering rapid deforestations due to the expansion of agricultural lands, the increasing demand of wood products, energy, and massive developmental projects, as well as natural disasters [2]. In the event that emissions caused by deforestation continue to occur in the coming decades, the Earth will become more vulnerable to the various negative impacts of climate change. The impacts will cause an inconsistent carbon balance, the loss of biodiversity, alteration of water regulation, and variation in weather patterns. Reducing emissions from deforestation and forest degradation in developing countries (REDD+) is one of the global initiatives that aims at conserving forests, enhancing carbon stocks and reducing carbon emissions [3]. The basic concept of REDD+ is that governments, private sectors or individuals who care for forests should be rewarded for conserving their forests instead of harvesting them for economic purposes. In this manner, it is expected that the initiative will contribute indirectly to the conservation of biodiversity, reducing habitat loss and ensuring the ecosystem will provide services as normal. Therefore, this option is considered the best method to maintain forests or to produce on a sustainable basis.

The REDD+ mechanism is based at the national level and therefore, the reporting should be at the national level. It is very important to have a national REDD+ scheme with a reliable, credible mechanism of measuring, reporting and verifying (MRV). Efficient approaches to providing accurate figures for emissions and removal from deforestation and forest degradation at the national level are necessary to make the REDD+ implementation successful. Its implementation also relies fundamentally on systems to assess available carbon stock and monitor changes due to loss of biomass from deforestation and forest degradation [3]. Therefore, efforts to provide reliable information on the extent of carbon stocks over particular forest areas, as well as to investigate their changes, have been progressing in many parts of the world, especially in tropical Asia [4]. Since Malaysia is in the phase of readiness for the implementation of REDD+ , there is an increasing need for such information.

Aboveground biomass (AGB) comprises all living components of vegetation (i.e., stems, branches, bark, seeds, leaves and undergrowth) that reside above the ground. Generally, 47% of the AGB composition is carbon (C) [5]. Usually, AGB is measured in metric tons of dry matter per hectare (e.g., t ha^−1^ or Mg ha^−1^) or in metric tons of carbon per hectare (e.g., t C ha^−1^ or Mg C ha^−1^). The higher the AGB in a forest, the higher the carbon stock that is stored in the forest. The United Nations Framework Convention on Climate Change (UNFCCC) identified AGB as an Essential Climate Variable (ECV). Therefore, methods for estimating forests AGB and carbon stocks need to be robust, consistent and transparent, to produce reliable estimates with minimal uncertainties [6].

Recently, remote sensing has been widely used for this process, and it was recognized as an essential tool for providing spatial information of forests [7, 8]. Remote sensing has been widely tested to map and monitor deforestation and has been suggested as primary tool for MRV. However, although remote sensing has been proven to be an effective tool, there is a debate about cost–benefit of using this technology. Remote sensing is somehow very subjective and depends on a wide range of ecosystem and land uses, scales of areas being monitored, as well as the approaches for accounting carbon credit [9]. For example, in Malaysia, the presence of clouds in remotely sensed data often hinders deforestation activities using optical sensor systems. It is almost impossible to acquire seamless images without clouds from optical systems, even if the images were collected within a 3-year timeframe [10]. Hence, SAR data become the sole possible approach for providing seamless images without clouds, within a given timeframe. This is because SAR systems have the advantage of penetrating clouds and are independent of light conditions. Because of this distinctive feature, SAR data are very useful for monitoring purposes, whereby consistency is the most crucial element in data acquisition. Therefore, the applications of SAR data for forest cover identification, deforestation detection and forest monitoring have been explored extensively in recent years. To some extent, SAR data are also used for landuse classification, forest stratification, stand and canopy structure studies and other biophysical predictions, including AGB [11].

This study used the Japanese Advance Land Observation Satellites (ALOS), also known as DAICHI, and ALOS-2 (DAICHI-2). Both carry Phase Array-type L-band SAR (PALSAR and PALSAR-2) sensors onboard that are useful for studying the biophysical properties of forests, including their changes over time [12, 13]. It was demonstrated that the sensitivity of PALSAR polarimetry depends on the structure, density, and tree elements (i.e., trunk/stem, branches, and leaves) of the forests [14]. PALSAR has shown better potential in retrieving the AGB of forests, including those in the tropics [15–19]. However, previous studies have demonstrated that AGB estimation using PALSAR system remains a challenging task, especially in tropical regions, with complex structures and unique conditions [20]. The presence of uncertainties in AGB estimations are due to (i) signal saturation at high AGB densities, (ii) a lack of, or limited ground-surveyed data, and (iii) the application of inappropriate image processing techniques. Efforts and scientific evidence have been devoted to understanding and identifying major uncertainties in AGB estimation. Therefore, this study proposed an approach for reducing these uncertainties and consequently addressed issues related to the mapping of forest cover, AGB estimation, quantification of changes to carbon stocks and, and thus, estimation of CO_2_ emissions (and removal) in a dipterocarps forests of Peninsular Malaysia. The study used multi-temporal datasets of PALSAR, which were acquired in the years 2007, 2008, 2009, 2010, and PALSAR-2, which were acquired in the years 2015 and 2016, to estimate AGB over the lowland, hill and upper-hill dipterocarps forests in Peninsular Malaysia. The estimation was carried out by integrating the PALSAR and PALSAR-2 images with a number of sample plots of AGB that were measured on the ground.

These time-series maps provide spatially explicit scenarios of changes in AGB over the time periods of this study. This information is expected to aid MRV for the REDD+ implementation in Malaysia and to stimulate the development of AGB maps for the other regions of Malaysia (i.e., East Malaysia). This study is in-line with the national needs and was conducted to identify advantages of the time-series of PALSAR and PALSAR-2 datasets in AGB, carbon stock changes, and CO_2_ dynamics of a large forest landscape [12]. The study is anticipated to contribute greatly to the improvement of current forest management, policy and governance.

## Methods

**Fig. 1:**
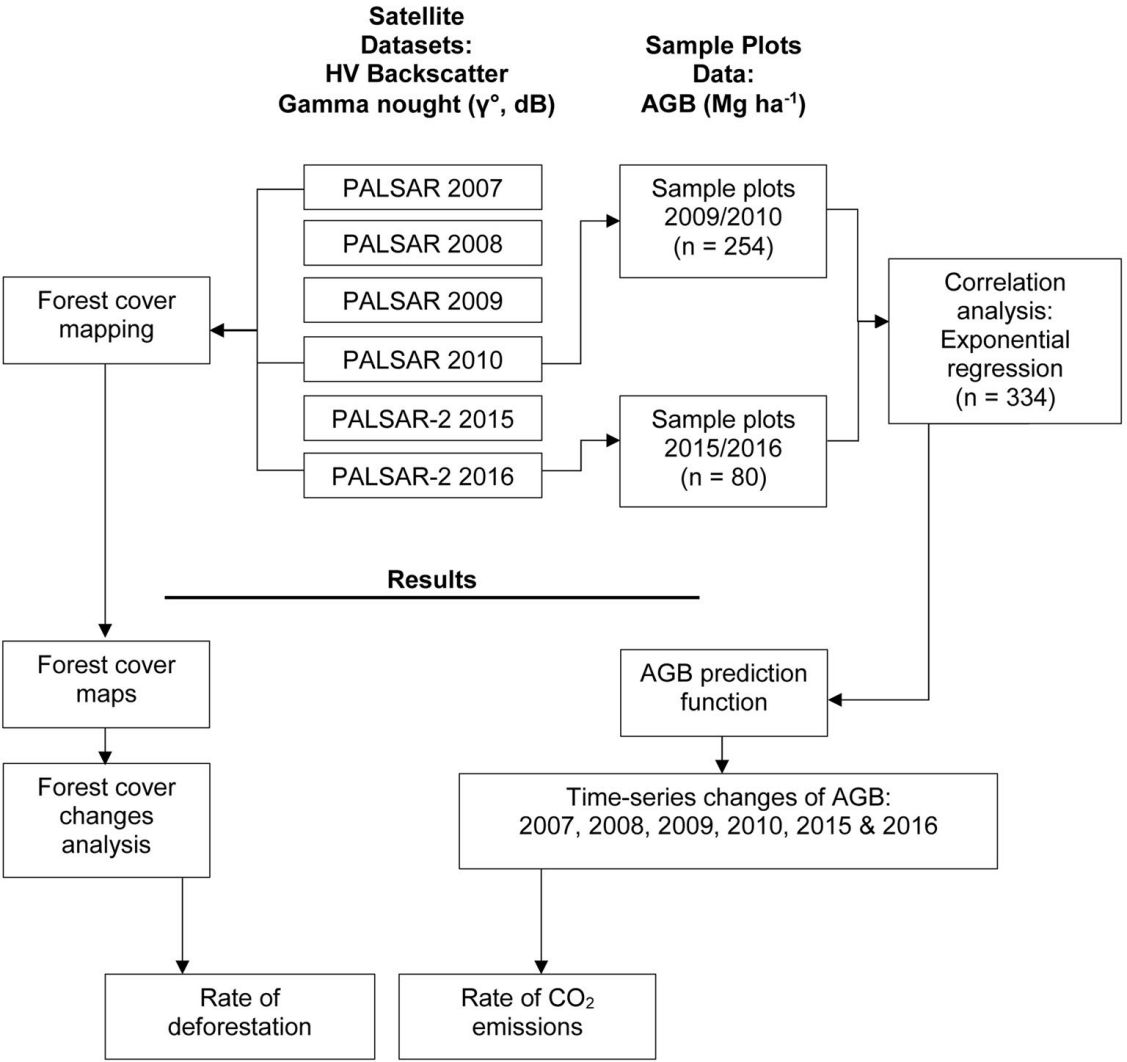
Flowchart of the methodology

The three major steps involved in this study are forest cover mapping, correlation analysis between satellites and field data, and analysis of time-series changes for the estimated AGB. Two main datasets that were used to execute these processes are mosaic products of PALSAR and PALSAR-2 and the AGB data that were measured in the field. Figure 1 shows the flowchart of the study.

### The study area

**Fig. 2:**
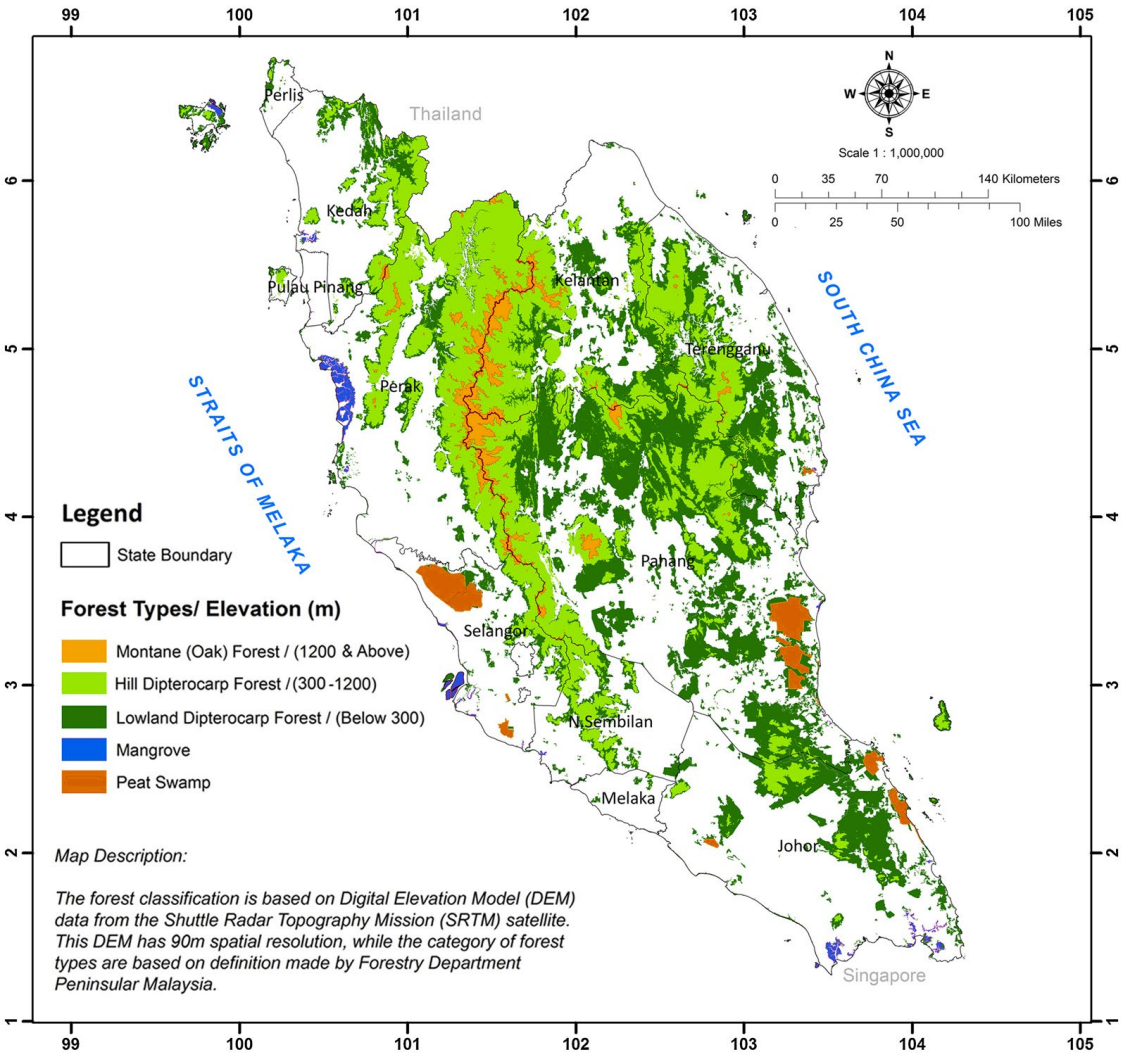
Major types of forests in Peninsular Malaysia

Peninsular Malaysia, which is located between 1–7° latitude and 99–105° longitude, generally has three major types of forest. Namely, these are inland, peat swamp and mangrove forests. These forests cover approximately 5.9 million ha or approximately 46% of the total landmass of Peninsular Malaysia. Inland forest is the major forest type and it comprises lowland dipterocarps (< 300 m a.s.l), hill dipterocarps (300–750 m a.s.l), upper-hill dipterocarps (750–1200 m a.s.l) and montane forests (> 1200 m a.s.l.). The forests cover approximately 93.5% of the total forested areas in Peninsular Malaysia. This study concentrates only within lowland dipterocarps, hill dipterocarps, and upper-hill dipterocarps forests, which cover some 5.26 million ha or 89% of the total forested areas in Peninsular Malaysia. These forests embrace all primary forests of the well-drained plains, undulating, and hill terrains up to 1200 m a.s.l. Tree species from the Dipterocarpacea family are common in these forests and thus make the forests the primary timber production areas in Peninsular Malaysia. The forests also store a considerable amount of carbon stock. Some areas are conserved as National Parks, where all pristine and virgin stands can be found [21]. Common dipterocarps trees found in this forest are from the genera of *Shorea, Dipterocarpus, Anisoptera, Hopea, Dryobalanops, Neobalacarpus,* and *Vatica*. Figure 2 shows the locality of major forest types found in Peninsular Malaysia.

### Satellite datasets

#### Satellite images

Datasets from (i) the Advanced Land Observing Satellite (ALOS) and (ii) ALOS-2 were used in this study. Phased Array type L-band Synthetic Aperture Radar (PALSAR) and PALSAR-2 are carried onboard ALOS and ALOS-2, respectively. The PALSAR and PALSAR-2 global mosaic datasets are created by the Japan Aerospace Exploration Agency (JAXA) by assembling adjacent satellite observation paths over extensive regions to form a seamless global mosaic. Corrections of geometric distortions specific to SAR (ortho-rectification), as well as topographic effects on image intensity (slope correction) have been applied using the SRTM-90 Digital Elevation Model. Backscatter is corrected for incidence angle and is thus given as gamma naught (γ°). The mosaics are given in geographical (lat/long) coordinates, using the GRS80 ellipsoid, and provided in rectangular tiles, 1° by 1° in latitudinal and longitudinal direction. The pixel spacing is 0.8 arc seconds, corresponding to 25 m at the Equator [22].

Table 1 summarizes the properties of the mosaic product and these datasets are referred to as “activity data” that have been used in this study. The global mosaic data can be downloaded free of charge from JAXA at http://www.eorc.jaxa.jp/ALOS/en/palsar_fnf/fnf_index.htm.

**Table 1:** Properties of the PALSAR and PALSAR-2 mosaic product

Year of acquisition	Satellite	Sensor
2007	ALOS	PALSAR
2008	ALOS	PALSAR
2009	ALOS	PALSAR
2010	ALOS	PALSAR
2015	ALOS-2	PALSAR-2
2016	ALOS-2	PALSAR-2

ALOS-2, carries the state-of-the-art of L-band SAR. Its mission is the follow-on of the ALOS mission and PALSAR-2 succeeds PALSAR onboard ALOS. ALOS PALSAR was launched in January 2006, and it was followed by ALOS-2 PALSAR-2 that was launched in May 2014. Both satellites are operated in a sun-synchronous orbit at 691 km, with a 46-day recurrence cycle. PALSAR-2 currently operates and produces L-band SAR data, which has similar (with some advancements) characteristics to PALSAR [23].

The SRTM-90 was also used in this study to acquire information on topography, which was used for forest type classification, according to the specified elevation categories. These data are produced by US Geological Survey’s Earth Resources Observation and Science (EROS) Data Center and it is available at http://srtm.usgs.gov/index.html.

#### Satellite image calibration

Mosaic products of PALSAR and PALSAR-2 are normally generated in a 16-bit data format. Each pixel on the mosaics was assigned by digital numbers (DN) range from 0 to 65,535. However, the DN do not represent the backscatter values. Therefore, the linear backscatter values in the HH and HV images can be converted from image DN to γ°, and expressed in decibel (dB), by the following formula [22]:

1 $$\upgamma^\circ = 10\cdot log_{10} \left( {DN^{2} } \right) - 83 [dB]$$

### Forest cover mapping

Forest cover classification was performed on the images to delineate the forests from other land cover types. This process was performed to create clear-cut boundaries of forest cover so that the estimation of AGB is not influenced by other features on the images. Radar backscatter from the canopies of forests is normally different from other vegetation and agricultural crops. However, it is often confused with rubber plantations because the backscatter from this crop is identical to that from forest canopies on both HH and HV polarizations [11]. To overcome this problem, several image variables were produced from the original HH and HV polarizations. The image variables, namely (i) simple band ratio (HH/HV), (HV/HH), (ii) average (HH + HV/2), (iii) square root of products (√(HH × HV)), and Gray-level co-occurrence matrix (GLCM), were produced.

The GLCM is often used in land use/land cover classification as a texture measure to improve classification. Texture is defined as a repeating pattern of local variations in image intensity, which is too fine to be distinguished as a separate class at the observed resolution. Therefore, a set of pixels having similar gray-level properties that occur repeatedly in an image region will constitute a texture. In this study, a mean-type GLCM was applied to the original images of both from HH and HV polarizations. This process produced textured images, with clearer definitions of the objects on the images [24].

These inputs were used in the classification iterations, and the Maximum Likelihood Classifier algorithms with nearest neighbor technique was applied. The forest cover was then divided into several forest types, according to the specified elevation using the DEM from SRTM. These processes were applied only on the images over the years 2007, 2010 and 2016 because the forest cover did not change significantly within a short period, i.e., 1 to 2 years. The results were used to quantify the rate of forest change, as well as to identify causes of deforestation that have occurred within the study periods.

### Field sampling

**Fig. 3:**
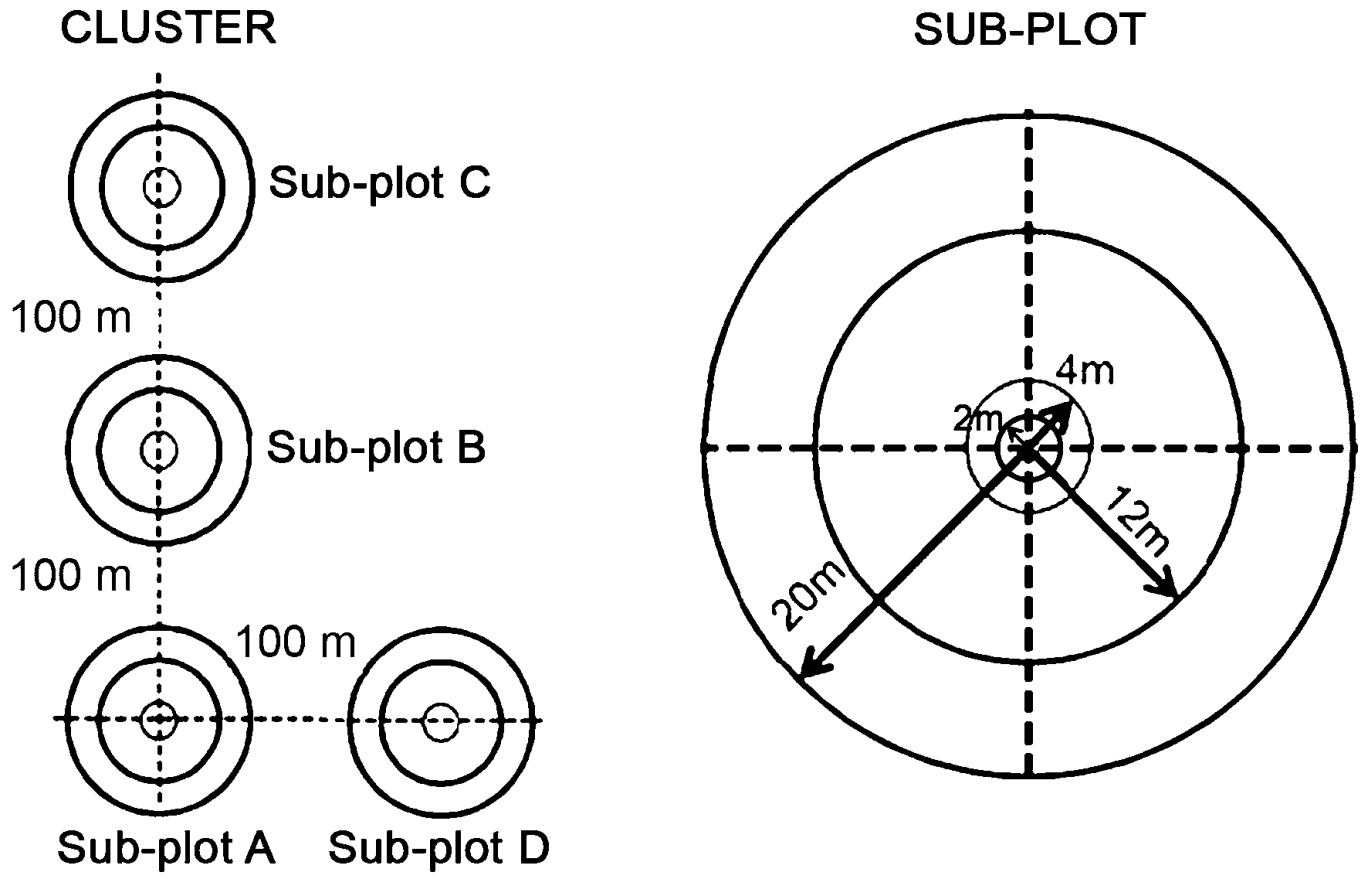
A cluster that comprises of four sub-plots and the design of a sub-plot

Principally, the standard operating procedure (SOP) for the sampling design was developed by Winrock International [25]. However, some modifications have been made on the design to suit the conditions of forests in Peninsular Malaysia. The SOP follows Intergovernmental Panel on Climate Change (IPCC) standards and is very practical for implementation in the field for AGB measurements [5]. A cluster is made up of at least four sub-plots, which are circular with several levels of nests inside, as shown in Fig. 3. The outermost nest measures 20 m in radius, followed by the smaller nests, measuring 12 and 4 m. Trees were measured according to the nest sizes as summarized in Table 2. The smallest nest, measuring 2 m in radius, was created to sample small trees or saplings. In this nest, all trees found within the radius were numerically counted but their diameter was not measured. This design indicates that not all trees were measured in a single plot because it depends on the nest size, which reduced the time spent in the field, while maintaining the sampling adequacy and representativeness for AGB estimation in a particular forest stratum.

**Table 2:** Sizes of trees that were measured in all nests in a sub-plot

Nest radius (m)	Size	Diameter at breast height, DBH (cm)
2	Sapling	DBH < 10 cm; height > 1.3 m
4	Small	10.0–19.9
12	Medium	20.0–39.9
20	Large	≥ 40.0

A natural forest ecosystem usually has five carbon pools, which are; (i) aboveground biomass of living trees, (ii) belowground biomass of living trees, (iii) deadwood and woody debris, (iv) non-tree vegetation and litter falls, and (v) soil. Among all of these carbon pools, the aboveground biomass of the trees constitutes the major portion of the carbon pool and it is directly affected by deforestation and forest degradation. Aboveground biomass comprises all the living components of a tree, including stem, branches, and leaves. Allometric functions, which typically include DBH and/or height, are the best way to estimate the AGB. In this study, an allometric function that was published for dry inland forests in the Asia region was used to estimate the AGB [26].

2 $${\text{AGB }} = \, [{ \exp }\left( { - 1. 80 3- 0. 9 7 6E + \, 0. 9 7 6ln\left( \rho \right) \, + { 2}. 6 7 3ln\left( D \right) - 0.0 2 9 9\left[ {ln\left( D \right)} \right]^{ 2} } \right]$$

AGB is aboveground biomass (kg/tree), *E* denotes bioclimatic variable, *ρ* represents wood density, and *D* is DBH.

**Fig. 4:**
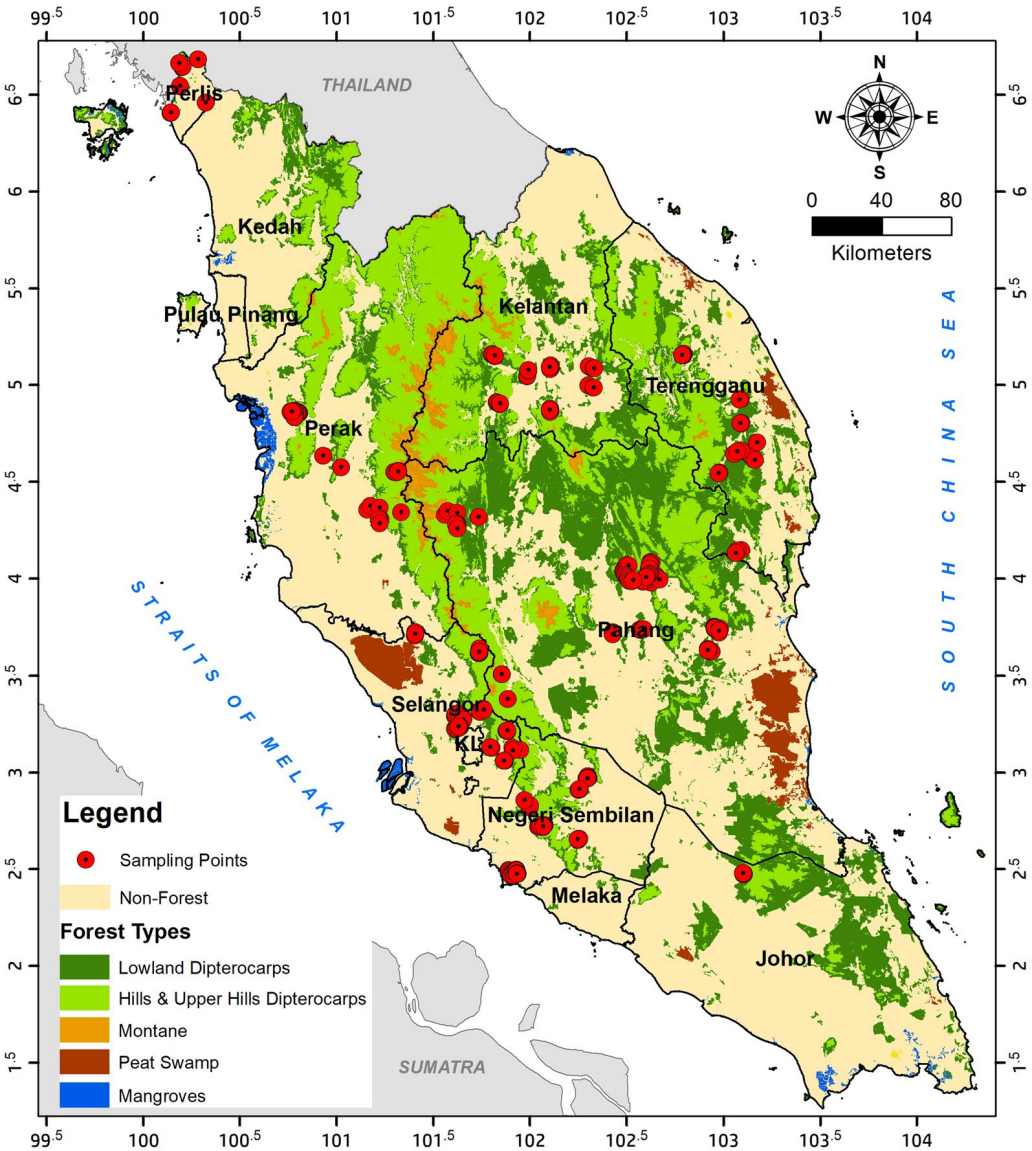
Distribution of sample plots within the study area

The forest survey was conducted in two phases: (i) phase 1, within years 2009–2010 and (ii) phase 2, within years 2015–2016. In the study, 334 plots were surveyed within two phases, and these were used as sample plots. Surveys were conducted during several trips, and the distribution of sampling plots was concentrated mainly at the central parts of Peninsular Malaysia, as shown in Fig. 4. The AGB within all sample plots ranged between 35.57 and 615.50 Mg ha^−1^, with an average of 399.42 Mg ha^−1^, as summarized in Table 3. The survey covered all conditions of forests, which are virgin (i.e., totally protected areas), natural forests (with good, moderate and low stand density), and logged forests to ensure that the final estimation was representative of all types and conditions of forests.

**Table 3:** Summary of the distribution of the sample plots within the study area

Phase	Year	Number of sample plot (n)	AGB (Mg ha^−1^)			
			Minimum	Maximum	Average	Std. dev.
1	2010	254	73.50	615.50	401.91	126.42
2	2016	80	35.57	596.37	392.37	131.10
1 and 2	2010 and 2016	334	35.57	615.50	399.42	127.63

### Aboveground biomass estimation

Since the forest surveys were conducted in two phases, i.e., within years 2009–2010 and 2015–2016, the correlations were generated based on datasets from 2010 (PALSAR) and 2016 (PALSAR-2). Backscatter values from HV polarization from both datasets were extracted from the images. The AGB values that were measured at the sample plots were correlated with the backscatter values at corresponding locations using a non-linear regression method. Several prediction functions have been produced, and the most representative function was used to retrieve AGB over the entire study area. The derived prediction functions used AGB as a dependent variable and the backscatter as the independent variable. Exponential functions are commonly applied to create this relationship, which is represented as *y *=* a.e* ^*b(x)*^, where *x* and *y* = HV polarization and AGB, respectively; *a* and *b* = model coefficients.

To check the accuracy of the estimates, root mean square error (RMSE) was calculated. In this case, the accuracy is a measure of the error between a derived/predicted AGB from the PALSAR and PALSAR-2 images and the actual AGB measured on the ground. The calculation can be expressed as follows:

3 $$RMSE = \varvec{ }\sqrt {\mathop \sum \nolimits \frac{{(AGB_{p} - AGB_{r} )^{2} }}{n}}$$

where RMSE is the root mean square error of the estimated AGB (± Mg ha^−1^), AGB_p_ and AGB_r_ are the predicted and reference AGB, respectively, and n is the sample size (i.e., number of sample plots).

In additional to the RMSE, the accuracies of the estimates were also measured in terms of percentage (%) using the mean absolute percentage error (MAPE), which can be calculated using the following formula.

4 $$MAPE = \left[ {\frac{1}{n}\mathop \sum \nolimits \frac{{AGB_{r} - AGB_{p} }}{{AGB_{r} }}} \right] \times 100$$

### Validation of the AGB estimates

The prediction function that was used to estimate AGB in the entire study area was validated using the K-fold cross-validation method, which is a technique that is used to evaluate a predictive model by dividing the original sample into (i) a training set to train the model and (ii) a test set to evaluate the model. The cross-validation process is then repeated k times (the folds), with each of the k subsamples used exactly once as the validation data [27]. In this study, tenfold cross-validation method was used where all sample plot data were randomly grouped by tens (10). This method was used because it can indicate the performance of a prediction model better than the common residual method. This method also provides an indication of how well the model makes new predictions over new sample data. One group was used as a testing set, while the other nine groups were used to develop the model. The RMSE was calculated using the testing set. This process was iterated 10 times and each group was used as a testing set once. Then, all RMSE were averaged to obtain an overall RMSE of the validated model.

### Time-series changes of AGB

Since the prediction equation has been developed from two sets of data (i.e., PALSAR 2010 and PALSAR-2 2016), the one that was derived from the combination of these data was used. The equation was applied to all images to produce estimates of AGB for each respective year, as listed in Table 1. Spatially explicit maps of AGB have been produced and the magnitude of AGB changes were quantified. Trends in AGB and carbon stocks changes were also observed. The AGB was converted to carbon stock (C) by multiplying the values with a constant factor of 0.47 [5]. Subsequently, the rate of carbon loss was determined throughout the period using the ‘activity data’. In this case, the activity data refers to information on the changes of forest cover that has been produced from the time-series estimates.

### CO_2_ emission and removal

The term CO_2_, known as carbon dioxide, is defined as a natural, colorless and odorless greenhouse gas that is emitted when fossil fuels (i.e., natural gas, oil, coal etc.) are burned. In this study, the CO_2_ emission is expressed as C loss, assuming that the gas is emitted when deforestation occurs. The unit of metric tons C was converted to CO_2_ by multiplying the ratio of the molecular weight of carbon dioxide to that of carbon (44/12 = 3.67) [5]. CO_2_ removal is different from CO_2_ reduction because the CO_2_ removal reduces emissions to the atmosphere but cannot reduce the amount of carbon dioxide already in the atmosphere. CO_2_ removal creates negative emissions, offsetting emissions from other sources such as domestic heating systems, airplanes and vehicle exhaust.

Generally, there are two methods that can be used to measure CO_2_ emissions resulting from deforestation. These are (i) the stock-difference method, and (ii) the gain–loss method. The stock-difference method requires two measurements in two epochs. The total change of carbon between these two epochs is calculated by dividing the total carbon stock changes of a particular unit area by the total period of the assessment. This method is the most suitable for calculating emissions from deforestation. On the other hand, the gain–loss method simply measures the mean annual increment of carbon in a particular area. Hence, this method is better suited for calculating emissions from forest degradation. Using the stock-difference method, net emissions from land use changes can be calculated based on following equation [5]:

5 $$\Delta C = \varvec{ }\frac{(C_{t2} - C_{t1})}{(t_{2} - t_{1})}$$

where ∆C is the rate of carbon stock change (Mg C year^−1^), and C_t1_ and C_t2_ are carbon stock (Mg C) at time t_1_ and t_2_, respectively. In this case, the ∆C was determined from the serial changes that have been produced from the activity data.

## Results and discussion

### Forest cover maps

Forest cover mapping was carried out on the images to produce classified images, with delineated forests from other land cover types. This process was carried out on images over the years 2007, 2010 and 2016, to produce different sets of forest cover maps that enabled the detection of changes and the identification of factors of deforestation. The study revealed that the HV polarization was very useful for delineating forest cover, similar to previous studies that were previously reported [28]. On the other hand, the HH polarization was effective in delineating crops and plantation areas, such as oil palm, rubber and teak. The HH is more sensitive to orientation than the HV; therefore, systematically planted trees and homogenous crops are well-interpreted on HH polarization. Table 4 summarizes the classification results that were produced using images over the year 2016. The estimation of AGB in this study included lowland, hill, and upper hill dipterocarp forests, which covered 5257,395 ha. The classification results were verified with land use maps for the years 2006, 2010, and 2014 that were produced by the Department of Agriculture Peninsular Malaysia. All classification results attained between 80% and 83% accuracies, with kappa coefficients of 0.77–0.81. The presence of classifications errors was attributed to the misclassification of rubber plantations and secondary forests, as these classes were defined differently on the land use maps, which were used as a reference. However, the errors did not affect the classification results, as the other land uses were neglected in this study.

**Table 4:** Extent of the study area during the year 2016

Forest type	Extent (ha)	Percentage (%)
Lowland dipterocarps	2,704,816	51.4
Hill dipterocarps	2,004,991	38.2
Upper-hill dipterocarps	547,588	10.4
Total	5,257,395	100

**Fig. 5:**
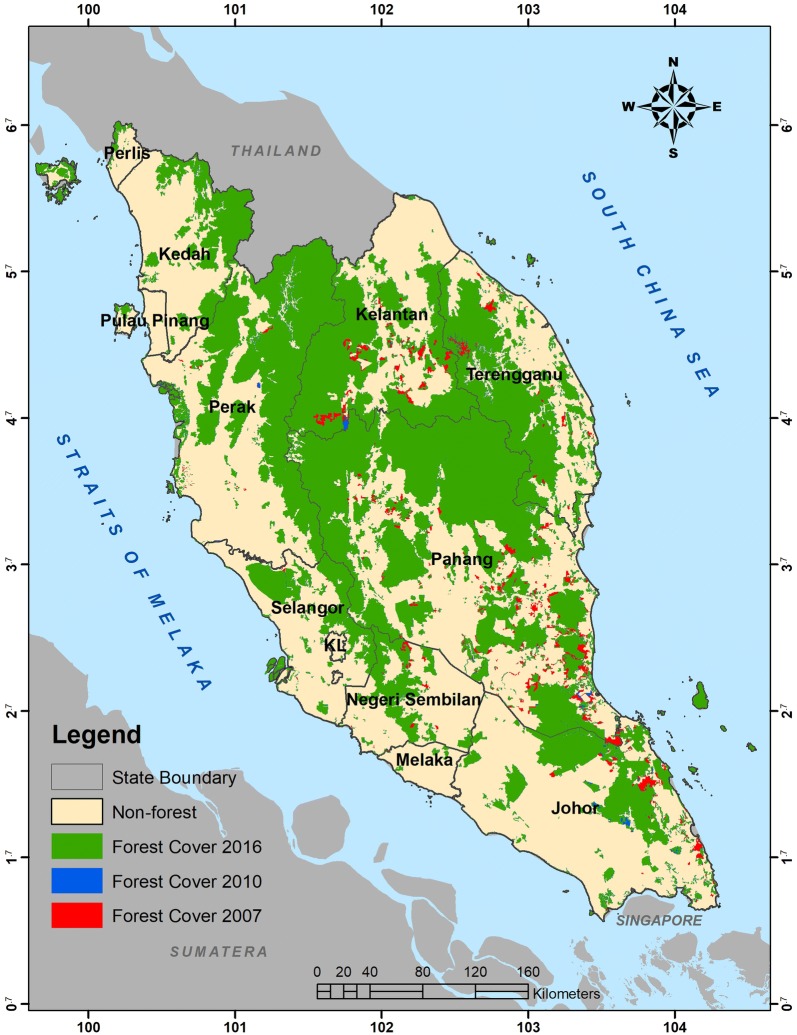
Deforestation activities within years 2007, 2010 and 2016

**Fig. 6:**
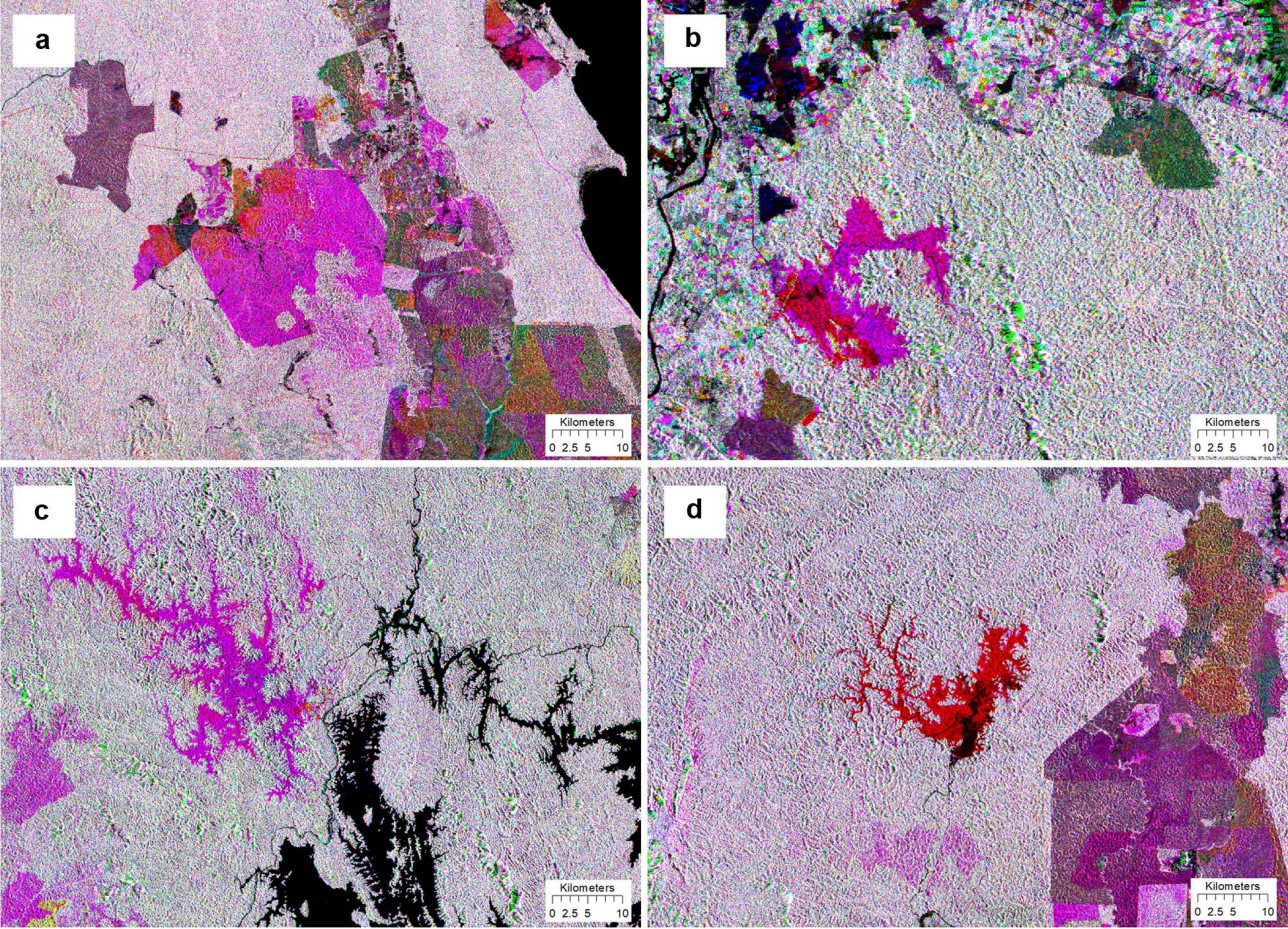
Color composites of PALSAR and PALSAR-2 images displayed in RGB = 2007, 2010, and 2016. The expansion of oil palm plantation occurred in year 2010 turns purple (**a**) and rubber plantation in years 2010 and 2016 turns red and purple, respectively (**b**). Hydroelectric dam reservoir that was constructed between 2010 and 2015 turns purple in (**c**), and hydroelectric dam reservoir that was constructed before 2010 turn red (**d**)

These maps were used to assess changes of forests within the study area. While Table 5 summarizes the magnitude of changes, Fig. 5 shows locations of the changes that have occurred within the period between 2007 and 2016. These changes were the consequence of deforestation or forests that were converted to other land uses. The study found that the total deforested area within the study area between 2007 and 2016 was 213,440 ha. The annual rate of deforestation within this period was quantified at 21,698 ha year^−1^ or 0.38% year^−1^. In this case, Peninsular Malaysia is considered a low deforestation country, with deforestation values of less than 0.5% year^−1^ [29]. Large pieces of forests in the region were converted to oil palm and rubber at commercial scales. In some other places, the forested areas were replaced by urban areas in terms of development, high density of human structures such as houses, commercial buildings, roads, and highways. Similarly, forests were converted to hydro-electric dams and reservoirs, as well as quarry and mine sites, from which mineral resources were excavated from the ground. Time-series images have greatly facilitated the detection process of changes because the dynamics of land uses can be observed easily on a color composite of multi-temporal data (Fig. 6). Direct causes of deforestation are usually linked to human activities that directly diminish forest cover and result in loss of C stocks. Agriculture was found to be the most common driver of deforestation worldwide and comprised 80% of all other drivers [30]. Similarly, commercial agriculture is the most prominent factor of deforestation in Asia and other tropical countries in Latin America and South Africa [31].

**Table 5:** Changes of the forest cover and rate of deforestation in Peninsular Malaysia

Forest cover (ha)			Changes (ha)	Rate of changes (ha year^−1^)	Rate of changes (% year^−1^)
Year 2007	Year 2010	Year 2016			
5,720,317	5,690,816	5,525,034	213,440	21,698	0.38

### Field sampling data

**Fig. 7:**
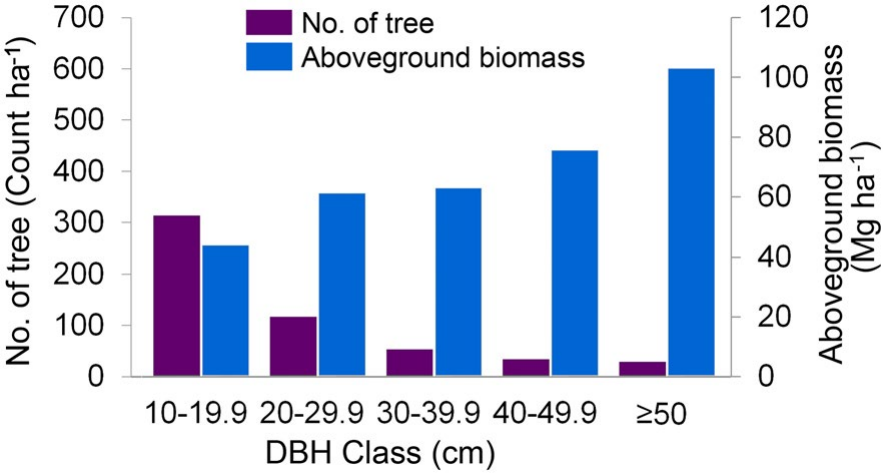
Average number of trees and AGB according to diameter classes in a dipterocarp forest

The study managed to collect a total of 334 sample plots covering the entire study area. Aboveground biomass was estimated at plot level, and it was found that the average AGB within the study area was 399.42 Mg ha^−1^. The AGB ranged between 35.57 and 615.50 Mg ha^−1^, with a standard deviation of 127.63 Mg ha^−1^. It is notable that the AGB of small trees (DBH 10–19.9 cm) contributed only approximately 15% of the total AGB in a hectare of dipterocarp forest. However, these trees are abundant in terms of number. Figure 7 shows the average AGB and number of trees in a hectare of the dipterocarp forest, according to diameter classes, and indicates that large portions of AGB actually belong to large trees. Although the number of large trees is typically low in a forest area, the quantity of AGB that is stored by these trees is large.

### AGB estimations

Backscatter values at corresponding sample plot locations were extracted from HV polarization on images over the years 2010 and 2016. Correlations analysis was carried out only on HV polarization, as it has proven to be the best polarization for AGB estimation [14, 32]. Generally, HV backscatter is dominated by volume scattering, which his beneficial for estimating AGB over the dense vegetation cover in a tropical forest. On the other hand, HH backscatter is dominated by volume-surface scattering and therefore it is not helpful for AGB estimation [33]. This forest also has high variability in canopy structure and surface roughness, which will give significant influence to the scattering, as observed by the cross-polarization term (i.e., HV). In this case, the forests that have been selectively logged, and which have very high variability in canopy structure, are theoretically observable by the HV polarization.

**Fig. 8:**
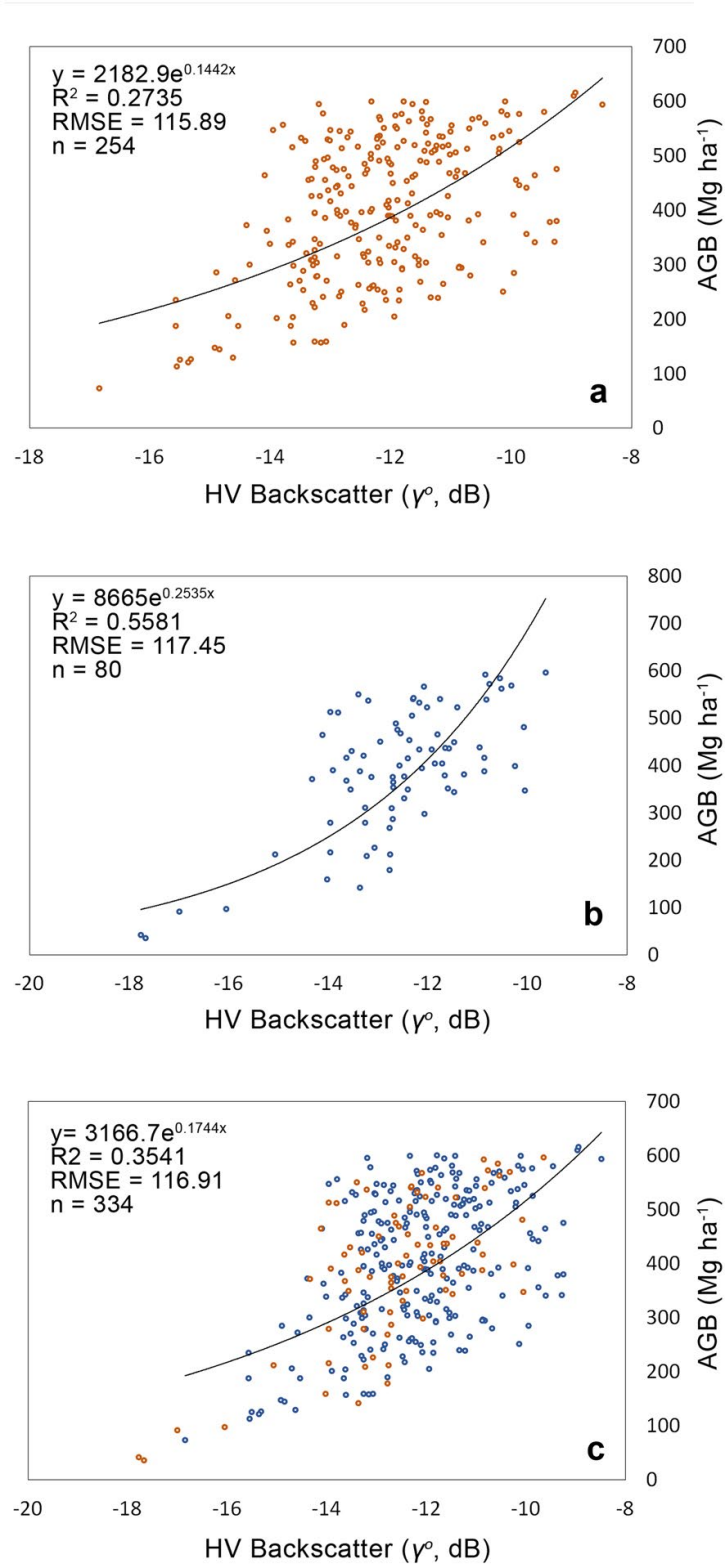
Scatter plots of correlations between AGB (*y*-axis) and backscatter (*x*-axis) over the year 2010 (**a**), the year 2016 (**b**) and the combined correlations (**c**)

The resulting prediction functions are summarized in Table 6, and the scatterplots are depicted in Fig. 8. Backscatter values from HV polarization over the sample plots ranged from − 8 to − 18 dB and tended to saturate at approximately − 12 dB [34–36]. The backscatter increased rapidly with AGB at low AGB levels (i.e., up to 200 Mg ha^−1^). However, the sensitivity started to decrease towards higher AGB levels (> 200 Mg ha^−1^) and eventually became almost constant afterwards, which is the point at which uncertainties exist in the AGB estimation, especially in tropical forests with an extremely high biomass [14]. This trend was similar for both correlations, generated from datasets over the years 2010 and 2016. However, the coefficient of determination (R^2^) from the 2016 datasets was higher than that of 2010, despite a smaller number of sample plots. Although the correlation was stronger, both datasets were combined to ensure that the estimation is representative and reliable of all forests types in the study area. The combined equation was used for AGB estimation over the entire study area and throughout all sample periods and can be written as

6 $$AGB = { 3166}. 7 {\text{e}}^{0. 1 7 4 4*HV}$$

where AGB is the aboveground biomass (Mg ha^−1^) and HV is backscatter (γ°, dB).

**Table 6:** Summary of the correlations between AGB and the backscatter of HV polarization

Year	Sensor	Prediction equation	R^2^	Number of samples (*n*)	RMSE (Mg ha^−1^)
2010	PALSAR	y = 2182.9e^0.1442x^	0.2735	254	115.89
2016	PALSAR-2	y = 8665e^0.2535x^	0.5581	80	117.45
2010 and 2016	PALSAR+PALSAR-2	y = 3166.7e^0.1744x^	0.3541	334	116.91

Backscatter is in gamma naught (γ°, dB) and AGB in (Mg ha − 1). All correlations are significant at p < 0.05

Since the sample plots covered only lowland, hill and hill dipterocarps forests, the equation produced was only valid for these forests and not accurate for other types of vegetation. This equation is also valid for the PALSAR and PALSAR-2 datasets. Nevertheless, the equation can be replicated and applied to other tropical countries that have similar forest physical characteristics.

Referring to Table 6, the RMSE that was obtained from the estimates was approximately 116–117 Mg ha^−1^. These figures reflect the threshold limit when estimating AGB from the L-band SAR data from PALSAR and PALSAR-2 in a tropical forest region such as Peninsular Malaysia. Meanwhile, the MAPE shows that the error of the overall estimates was 29.3%, which is equal to an accuracy of 70.7%. Therefore, the accuracy-error propagation was approximately 70–30, following IPCC good practice and guidance. The study revealed that this amount of error (i.e., approximately 30%) should be allowed when applying a pixel-based AGB estimation approach using L-band SAR data.

### Validation of the AGB estimates

**Fig. 9:**
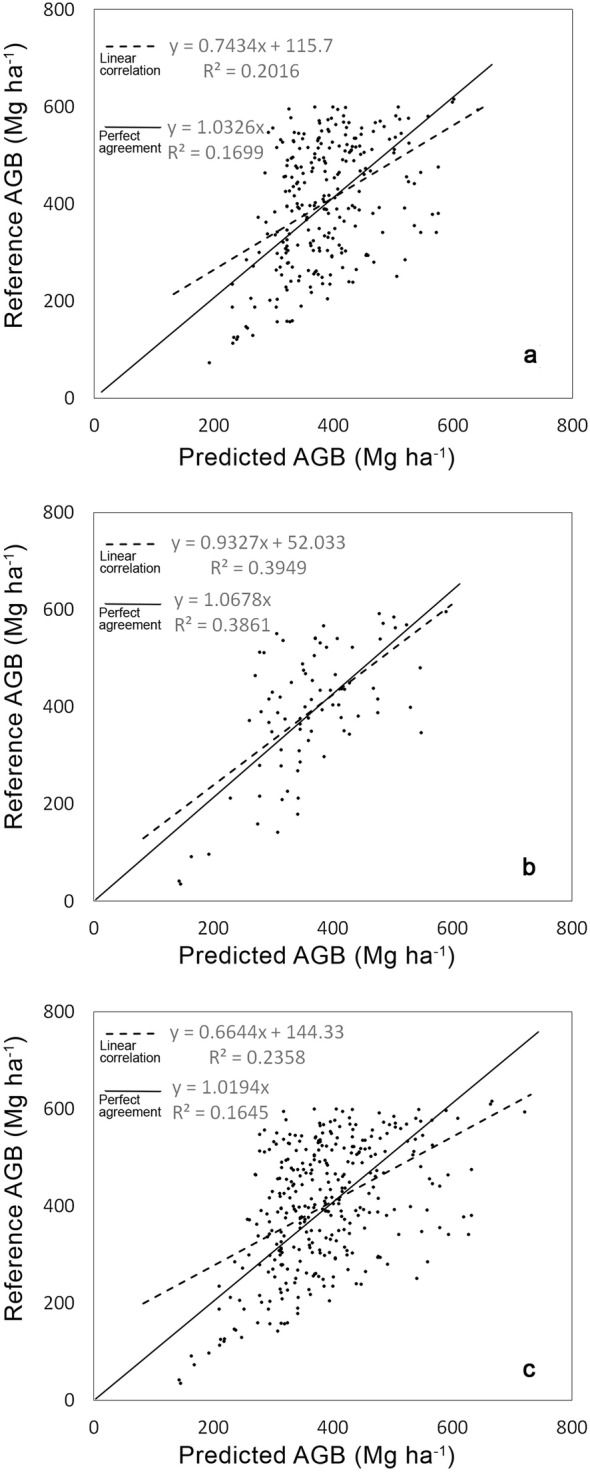
The agreements between reference and predicted AGB. Scatter plots show correlations between the reference AGB (y-axis) and the predicted AGB (x-axis) over the year 2010 (**a**), the year 2016 (**b**) and the combined models (**c**)

The validation method that was applied to the estimations has produced RMSE for each prediction function. The calculated RMSE for the prediction equations that were derived from PALSAR, PALSAR-2 and the combination of PALSAR and PALSAR-2 (Table 6) were 115.89, 117.45 and 116.91 Mg ha^−1^, respectively. Generally, each prediction function estimated the AGB within the study area at approximately the same level of accuracy, with small variations of RMSE. Prediction from a single sensor, i.e., either PALSAR or PALSAR-2, produced approximately the same results. However, only the one that was derived from the combination of PALSAR and PALSAR-2 was used to estimate AGB for the whole study area. Figure 9 shows the scatterplots generated from the reference and predicted AGB using tenfold cross validation. The scatterplots clearly show that the errors occurred largely at the lower-end and higher-end of the AGB levels than 400 Mg ha^−1^. In the other words, the prediction function has overestimated when the AGB is lower than 400 Mg ha^−1^ and underestimated when the AGB is higher than 400 Mg ha^−1^.

### Time-series change of AGB and C stocks

**Fig. 10:**
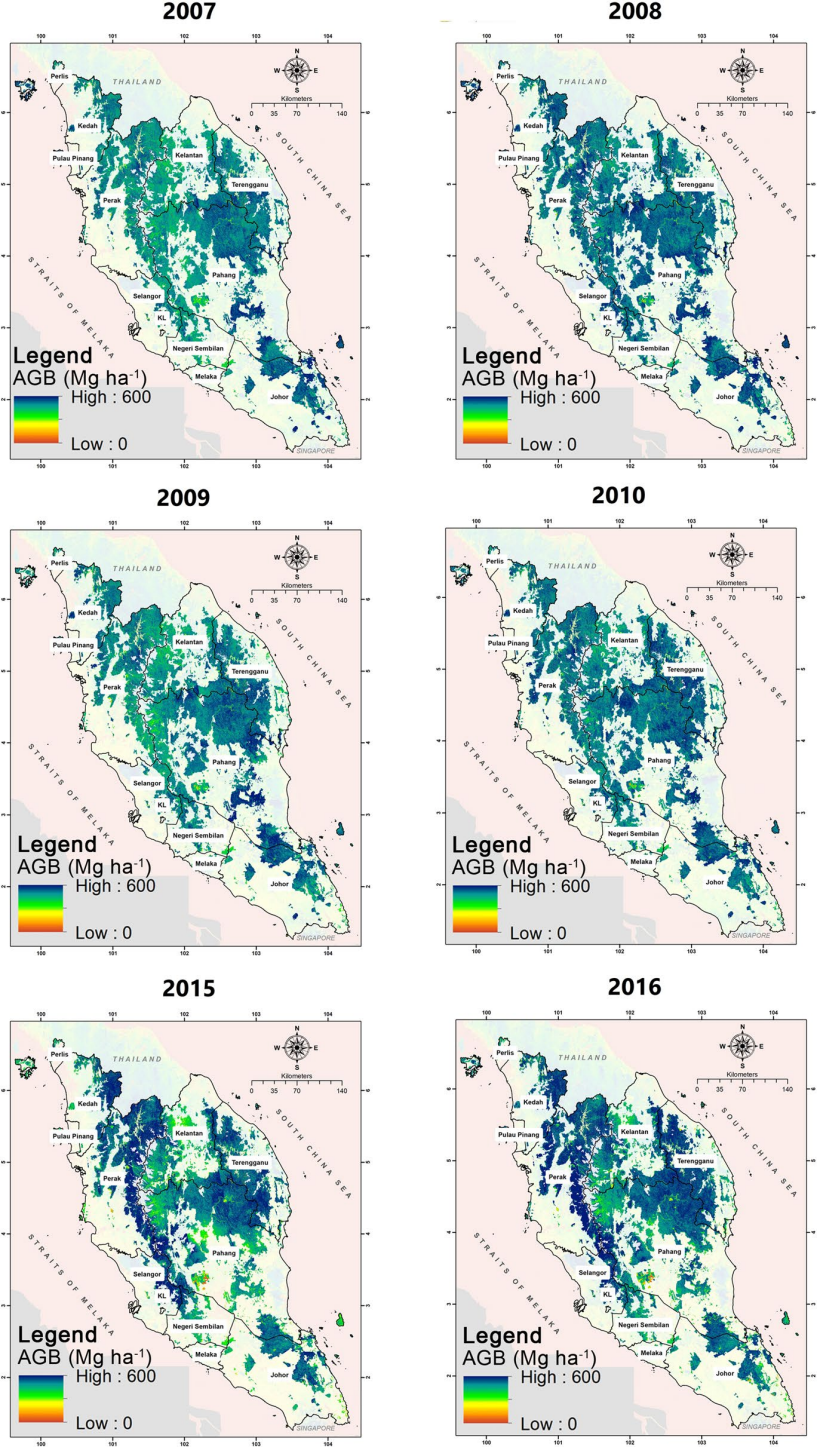
Spatially explicit maps of AGB over the study areas, on the years as indicated

**Fig. 11:**
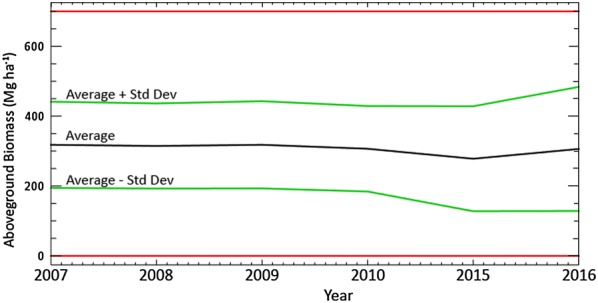
Basic statistic of time-series AGB in the study area

**Fig. 12:**
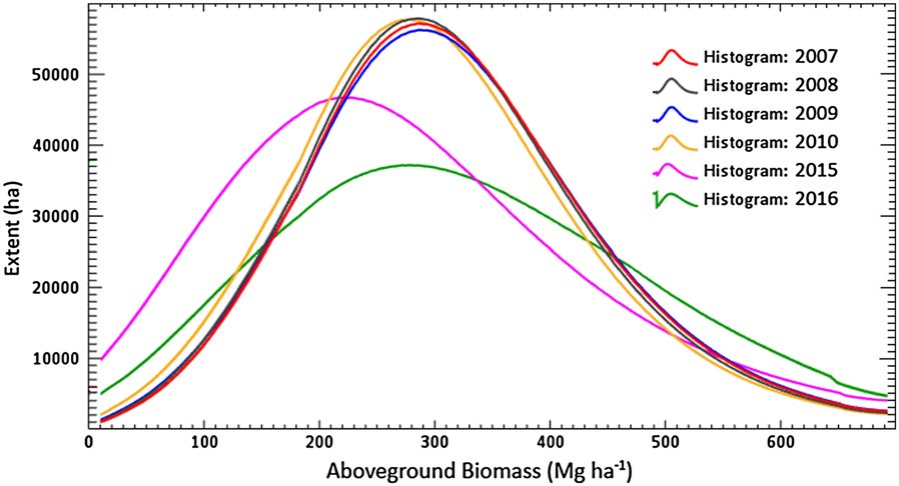
Histogram of time-series AGB distribution in the study area

The study produced spatially explicit maps indicting the distribution of AGB within the study for all series (Fig. 10). A high density of AGB dominated the central parts of Peninsular Malaysia. These areas are covered by virgin forests, where the largest national park is located within the borders of Pahang, Terengganu and Kelantan. High levels of AGB also occur along the Main Range, stretching the west coast of Peninsular Malaysia from Kedah to Negeri Sembilan. It is also notable that a high density of AGB occurs in the north of Perak, where the Royal Belum State Park is located. These forests are known to have existed a million years ago and remain intact as virgin forests. Variations of AGB levels are patchy and spread within the other areas, which consisted of natural, logged and secondary forests. A low density of AGB occurred along the outer edges of the forests, adjacent to other land uses. From these maps, the statistics of the distribution were extracted to observe the dynamics of AGB and thus, C stock changes within the study area. Basic statistics and histograms of the distribution are depicted in Figs. 11 and 12, respectively. It is obvious that the changes of AGB occurred significantly when the large deforestations took place within the study area. In the meantime, there were also some areas that indicated increments in AGB, which were the result of carbon sequestration, especially in totally protected areas.

**Fig. 13:**
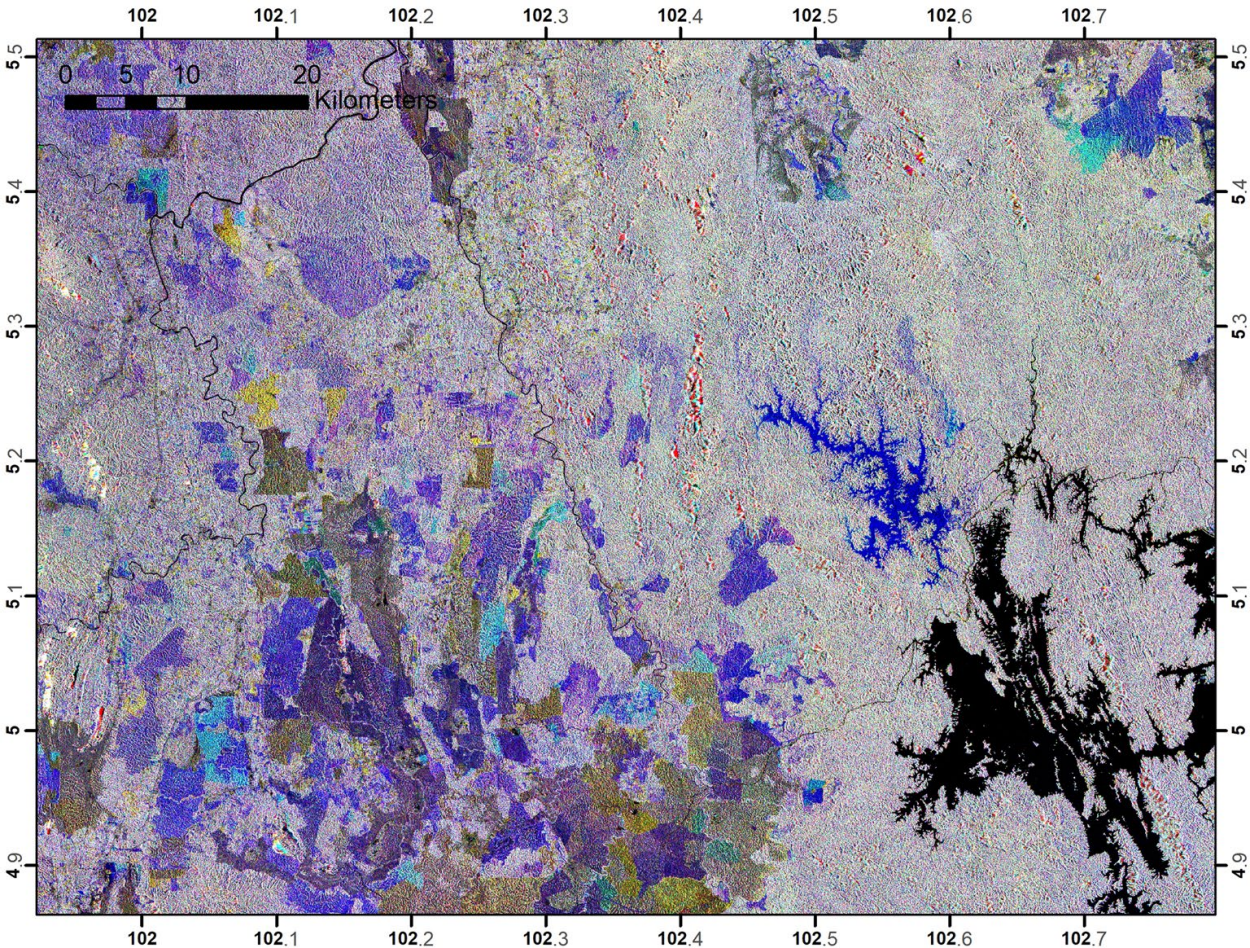
Color composite of AGB images, displayed in RGB = 2016, 2010, and 2007. The dark blue patches indicates clear-cut areas due to deforestation and lighter colors represent dynamics of AGB within the periods. Gray color indicates areas that have no change, i.e. the remaining forests, and the black patches are unchanged water bodies

The differences that exist in these statistics reflect the overall changes that have occurred within the study areas because they accounted for millions of pixels. Referring to Fig. 11, the mean AGB was almost constant from the year 2007 through 2010. However, the mean value slightly dropped in the year 2015 and increased again in 2016, which means that there was no significant deforestation or land use conversion activities occurring between 1-year differences from 2007 through 2010; however, these activities occurred significantly during the 5-year period between 2010 and 2015. This quantitative statistic was cross-checked qualitatively with a color composite of AGB maps, as shown in Fig. 13. Notably, when the AGB images were draped over each other, the deforested areas are visibly enhanced as consequent of the loss of C stocks [37, 38].

**Fig. 14:**
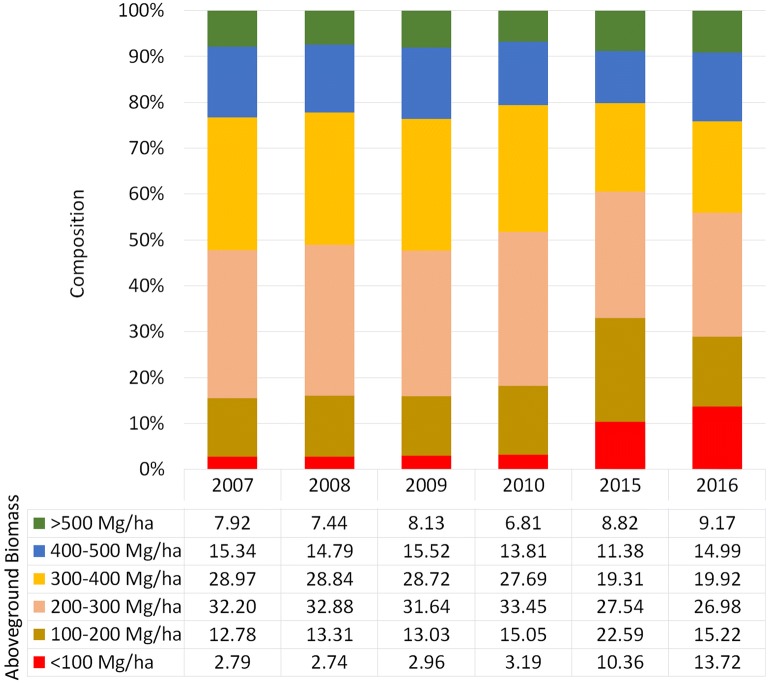
Summary of AGB distribution composition in time series

Similarly, effects of AGB reductions and sequestration can be observed through the extracted histogram as shown in Fig. 12 and AGB composition in Fig. 14. Apparently, the AGB within the range of 200–400 Mg ha^−1^ dominated the study area. The number of pixels that fall within low AGB values increased significantly in the year 2015. This pattern expresses areas that were previously (before 2015) covered by forests with high AGB density and were then cleared and replaced with other crops. During that time, the AGB became zero (0). After 1 year (2016), the number of zero-value AGB increased as the barren areas were replaced by other crops. These crops will continue to sequester carbon and thus increase the AGB. There were also some areas within the study area that were designated as forest plantations. These areas are deliberately cleared and then planted with specific timber trees for commercial purposes. Normally these areas involved a considerable extent of land and are visible on the AGB maps. This type of dynamic was reflected on the histogram because the boundary for the statistic extraction was from forest cover in the year 2007. Therefore, any change occurring after this was captured in the histogram.

**Fig. 15:**
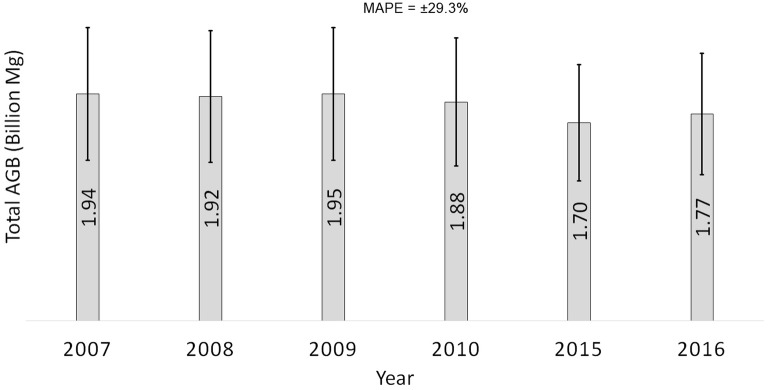
Total AGB in the study area accounted in time series

The total AGB fluctuated in the periods between the years 2007 and 2016. The reduction of total AGB was mainly due to deforestation and also extraction of timber in production forest. Out of 4.92 million ha of Permanent Reserved Forests (PRFs) in Peninsular Malaysia, 2.99 million ha is designated for production, where all timber extraction activities are conducted. Some 40,000 ha of this area is open annually for logging operation [21]. Although the harvesting is operated selectively, the extraction of timber will eventually reduce a considerable amount of existing AGB in those particular areas. However, as this forest is left untouched after logging, it will grow naturally and hence increase the AGB. The annual rate of carbon sequestration in dipterocarps forests was estimated to be approximately 3 Mg C ha^−1^ year^−1^ (or 6.4 Mg ha^−1^ year^−1^ of AGB) [39], which is actually the main reason for the pattern of AGB dynamics in Peninsular Malaysia. A drastic drop of the total AGB in the year 2015 was mainly due to permanent deforestation for the construction of hydroelectric dams. The forests in these particular areas have become permanent reservoirs, and there was entirely no biomass afterwards. This information is captured in the time-series maps of AGB and is translated into the total AGB, as reported in Fig. 15. Error bars on the top of the chart indicate the mean absolute percentage error of the estimations.

### CO_2_ emission and removal

**Fig. 16:**
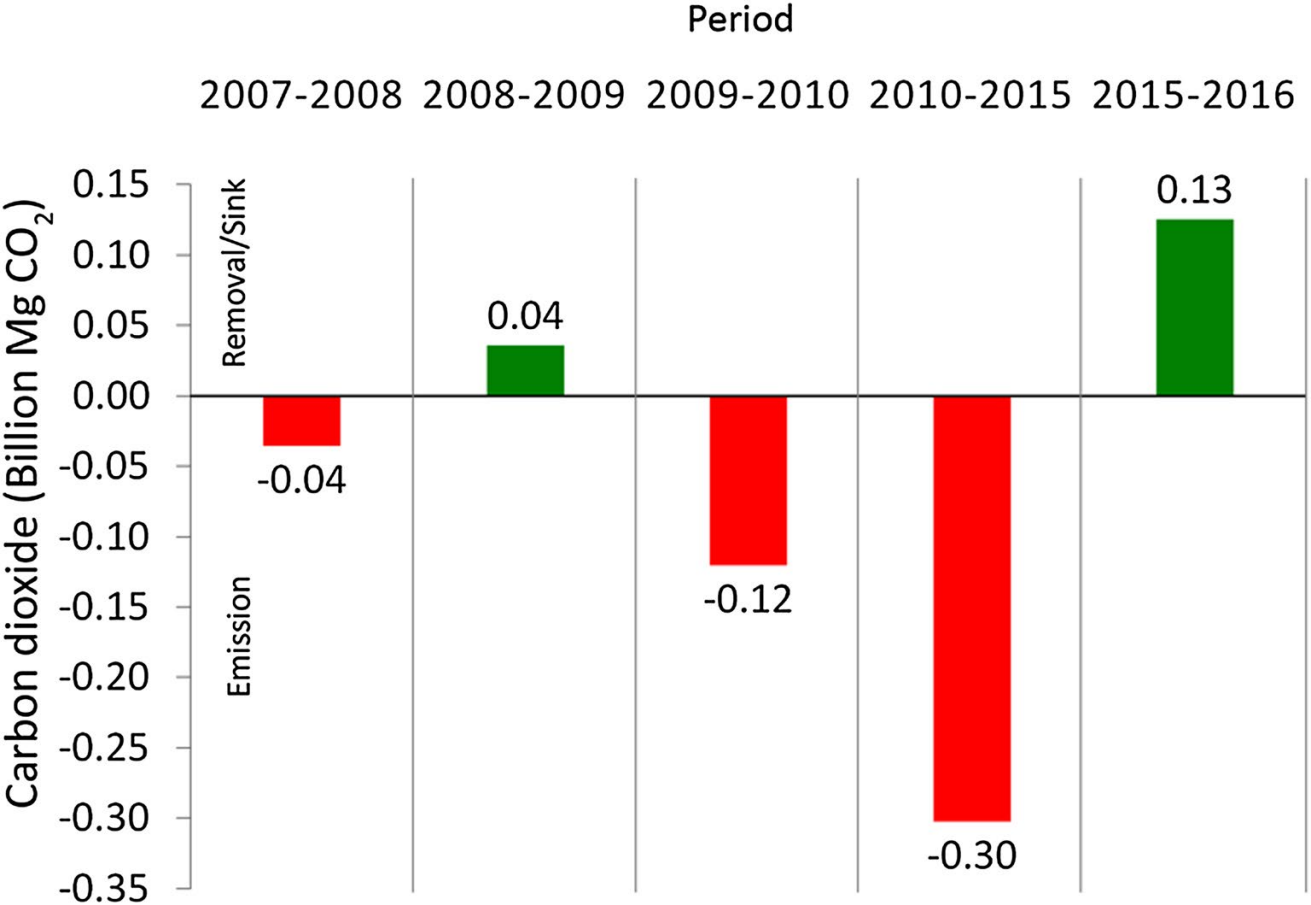
Net CO_2_ emission and removal from the activity data

Information that has been derived from the activity data was used to calculate and quantify changes of C stocks within the periods, as summarized in Table 7. The annual rate of change was also derived from the information. Subsequently, cumulative CO_2_ emissions between the years 2007 and 2016 were accounted for, as summarized in Table 8. Net CO_2_ emission was measured to be − 458,348,374 Mg CO_2_ and removal was + 161,301,555 Mg CO_2_. Somehow, the difference between the emission and removal has left approximately net CO_2_ emission of − 297,046,819 Mg CO_2_. According to this figure, the annual rate of emission was − 33,005,202 Mg CO_2_ year^−1^. An overall picture of the CO_2_ emission and removal cycle is shown in Fig. 16.

**Table 7:** Summary of time-series AGB and C stocks and rate of changes in the study area

Year	AGB (Mg)	Carbon stock (Mg C)	Total C change (Mg C)	Rate of C change (Mg C year^−1^)
2007	1,944,851,626	914,080,264	0	0
2008	1,924,372,988	904,455,304	− 9,624,960	− 9,624,960
2009	1,945,061,005	914,178,672	9,723,368	9,723,368
2010	1,875,228,104	881,357,209	− 32,821,464	− 32,821,464
2015	1,699,815,037	798,913,067	− 82,444,142	− 16,488,828
2016	1,772,640,588	833,141,077	34,228,009	34,228,009

**Table 8:** Net CO_2_ emissions and removal from the activity data

Net (Mg CO_2_)	Year					
	2007–2008	2008–2009	2009–2010	2010–2015	2015–2016	Total
Emission	− 35,323,603	–	− 120,454,771	− 302,570,000	–	− 458,348,374
Removal	–	35,684,761	–	–	125,616,794	161,301,555

Figure 15 generalizes the net CO_2_ emissions and removal that have been measured from the activity data from this study. There were consistent CO_2_ emissions and removal between the years 2007 and 2009, where emission activities in 2008 had been offset by CO_2_ emission removal in the year 2009, at approximately 0.04 billion Mg CO^2^. However, the deforestation and land use activities between the years 2009 and 2015 continued to emit CO_2_, without any offset. Sequestration in the year 2016 was eventually removed and the CO_2_ emission was approximately 0.13 billion Mg CO_2_. This pattern actually reflects the effectiveness of overall structural planning and management practices of the forests in Peninsular Malaysia. Information that has been produced by this study is valuable for the REDD+’s MRV and is important in the management and protection of forested areas.

## Conclusions

The study has successfully produced spatially explicit, time-series estimates of the AGB over the years 2007, 2008, 2009, 2010, 2015, and 2016, with an overall RMSE of approximately ± 117 Mg ha^−1^ at an accuracy at approximately 70%. According to these maps, the forests within the study area in are currently store approximately 1.77 billion Mg of AGB, which is equals to approximately 833 million Mg C, with an average density of 146 Mg C ha^−1^. The cumulative CO_2_ emission between years 2007 and 2016 was accounted at approximately ***− ***458 million Mg CO_2_ and removal at some + 161 million Mg CO_2_. Somehow, the difference between the emission and removal has left an approximately net CO_2_ emission at approximately ***− ***297 million Mg CO_2_. Prior to these estimates, the rate of deforestation has been determined at 0.38% year^−1^.

Although there were limitations found, this study provided an alternative for AGB retrieval that can be utilized in a practical manner to assist decision makers. This study, to some extent, can provide a significance contribution towards the MRV in the REDD+ implementation. An effective implementation of REDD+ activities needs to be based on a robust estimate of emissions, as presented in this study. MRV should follow the UNFCCC principles of transparency, consistency, comparability, completeness, and accuracy. This study met these requirements and the report presented was very clear. Therefore, L-band SAR remote sensing such as PALSAR and PALSAR-2 systems has been identified as a key technology to successfully implement and monitor a future REDD+ mechanism.

One of the greatest advantages of using these data is that the datasets are free to access. Free-cloud cover and rapid acquisition make them more valuable, especially for this type of study in Malaysia. Overall, the method that was presented here is applicable for achieving optimal goals, with reliable accuracy, low operational cost and very consistent available satellite data, which is very promising for the future.

## Abbreviation

ALOS: Advance Land Observation Satellites
AGB: aboveground biomass
C: carbon
CO_2_: carbon dioxide
DN: digital numbers
ECV: Essential Climate Variable
EORC: Earth Observation Research Center
EROS: Earth Resources Observation and Science
GLCM: Gray-level co-occurrence matrix
HH: horizontal-horizontal polarization
HV: horizontal-vertical polarization
IPCC: Intergovernmental Panel on Climate Change
JAXA: Japan Aerospace Exploration Agency
JERS: Japan Earth Resources Satellite
MRV: monitoring, reporting and verification
NRCS: Normalized Radar Cross Section
PALSAR: phase array type L-band synthetic aperture radar
REDD+: reducing emissions from deforestation and forest degradation
RMSE: root mean square error
SAR: synthetic aperture radar
SRTM: Shuttle Radar Topography Mission
UNFCCC: United Nations Framework Convention on Climate Change
